# HP1717 Contributes to *Streptococcus suis* Virulence by Inducing an Excessive Inflammatory Response and Influencing the Biosynthesis of the Capsule

**DOI:** 10.3390/microorganisms7110522

**Published:** 2019-11-03

**Authors:** Liang Liu, Qiang Zhang, Zhongmin Xu, Jingjing Huang, Weifeng Zhu, Anding Zhang, Xiaomei Sun, Meilin Jin

**Affiliations:** 1 Unit of Animal Infectious Diseases, National Key Laboratory of Agricultural Microbiology, College of Veterinary Medicine, Huazhong Agricultural University, Wuhan 430070, China; robin2371@163.com (L.L.); 05yisan@163.com (Q.Z.); xzmjack@126.com (Z.X.); zhuweifeng8@126.com (W.Z.); andye8019@mail.hzau.edu.cn (A.Z.); 2 Key Laboratory of Preventive Veterinary Medicine in Hubei Province, The Cooperative Innovation Center for Sustainable Pig Production, Wuhan 430070, China; sunxm320@126.com; 3 Key Laboratory of Development of Veterinary Diagnostic Products, Ministry of Agriculture, Wuhan 430070, China

**Keywords:** *Streptococcus suis*, inflammatory, streptococcal toxic shock-like syndrome, virulence, capsule

## Abstract

*Streptococcus suis* 2 (SS2) is an important zoonotic pathogen that substantially harms the swine industry and poses threats to human health. Excessive inflammation is considered to be a hallmark of SS2 infection because it is responsible for most clinical signs of SS2, especially streptococcal toxic shock-like syndrome. However, the current knowledge of SS2-induced excessive inflammation remains limited. In this study, we identified HP1717 as a novel extracellular pro-inflammatory protein in SS2 that can induce robust expression of inflammatory cytokines in RAW264.7 macrophages. Notably, the pro-inflammatory ability of HP1717 was dose-dependent and heat-sensitive, and it required the recognition of Toll-like receptor 2 (TLR2) and the phosphorylation of both extracellular signal-regulated kinases 1/2 (ERK1/2) and nuclear factor kappa-light-chain-enhancer of activated B cells (NF-κB). Further, by constructing a deletion mutant, we demonstrated that HP1717 significantly influenced the biosynthesis of the bacterial capsule, which plays a critical role in the virulence of SS2 by interfering with the ability of host immune cells to phagocytize and kill the pathogen. Indeed, the mutant strain displayed reduced resistance to whole-blood killing compared with the wild strain. Finally, murine experiments indicated that the deletion of *hp1717* in SS2 reduced the lethality, pro-inflammatory activity, and bacterial loads in mice. Collectively, our data reveal HP1717 as a novel virulence-related factor of SS2 that can induce an excessive inflammatory response and significantly affect the bacterial capsule, thus expanding our understanding of the pathogenesis of *S. suis*.

## 1. Introduction

*Streptococcus suis(S. suis*) is an important swine pathogen that is responsible for tremendous financial losses in the pig industry, and it presents a severe threat to human health [1]. Among the 29 described serotypes on the basis of capsular polysaccharide (CPS) antigens, serotype 2 (SS2) is frequently isolated from diseased pigs in most countries [2]. Since the first report on the pathogen was published in 1954, *S. suis* infection has been reported in more than 30 countries and regions [3]. Moreover, its prevalence in human populations has been increasing over the past 20 years. Between 2002 and 2013, there were 1642 cases of human infections in 34 countries, 90% of which occurred in Asia [1]. In China, *S. suis* caused two large-scale human infections in Jiangsu and Sichuan, infecting more than 240 people, 14 and 38 of whom died in 1998 and 2005, respectively [4]. Among the patients who died from the disease in 2005, 97.4% had streptococcal toxic-shock-like syndrome (STSLS) [5], and the mortality of patients with toxic shock was 62% [6]. Later, patients in Vietnam and Thailand presented similar symptoms [7], and these cases received considerable attention worldwide. Sequencing analysis showed that the two epidemics in China were caused by sequence type 7 (ST7) rather than the widespread pathogenic bacterial strain sequence type 1 (ST1) in Europe. Further investigation indicated that ST7 could stimulate the massive production of pro-inflammatory cytokines [8], which significantly contribute to the development of STSLS [9]. The results of these analyses indicated that Asia was facing a novel, highly virulent mutant strain of SS2 [10].

Previous studies have reported more than 100 virulence-related factors of SS2, such as subtilisin-like protease, muramidase-released protein, suilysin, and its CPS [11]. CPS is considered to play a particularly critical role in the virulence of SS2 because it can protect bacteria from host immune cells by preventing phagocytosis and bacterial death [12]. Despite these studies on SS2, the current understanding of its pathogenic mechanism remains limited, and the bacterial factors that lead to STSLS are particularly elusive [13]. STSLS was originally caused by group A *Streptococcus* infection and primarily associated with superantigens [14]. However, genome analysis showed that SS2 had neither superantigens nor homologous genes, suggesting that several unique molecular mechanisms could be responsible for STSLS due to SS2 [14]. A previous study indicated that excessive inflammation played a key role in the pathogenesis of SS2-induced septicemia, meningitis, STSLS, and sudden death [15]. The identification of several pro-inflammatory proteins (e.g., HP0459 and HP1330) has enhanced our understanding of STSLS [13,16], although this knowledge is not complete. Thus, further details on the mechanism of the excessive inflammation caused by SS2 will help us better understand SS2-induced STSLS.

In the current study, we identified the potent pro-inflammatory protein HP1717 in SS2 and studied its pattern recognition receptor and signal transduction pathway. We also demonstrated that the deletion of HP1717 in SS2 could significantly increase the whole-blood killing of the pathogen by affecting the biosynthesis of CPS. Our study not only reveals a novel virulence-related factor of SS2 but also considers, for the first time, both excessive inflammation and the bacterial capsule at the same time. Therefore, our results provide new insights into the pathogenesis of SS2 and further our understanding of this pathogen.

## 2. Materials and Methods

### 2.1. Bacterial Strains, Plasmids, and Growth Conditions

The details of the bacterial strains and plasmids used in this study are shown in Table 1. SS2 strain 05ZY was selected as the wild-type (WT) strain, which is highly pathogenic to mice and pigs and can cause STSLS. The 05ZY strain and the deletion mutant Δ1717 were cultured in tryptic soy broth (TSB) or on tryptic soy agar (TSA) plates (Difco, MI, USA) with 10% newborn bovine serum (Sijiqing Biological Engineering Materials Co., Ltd., Hangzhou, China) at 37 °C.

### 2.2. Cell Culture

RAW 264.7 macrophages were cultured in Dulbecco’s modified Eagle’s medium supplemented with 10% fetal bovine serum (Gibco, Waltham, MA, USA) at 37 °C in a 5% CO_2_ atmosphere. The cells were plated at a density of 10^6^ cells per well in 12-well plates. Mouse primary macrophage were isolated according to previous research [13]. Toll-like receptor 2 (TLR2)-deficient, TLR4-deficient, and WT mice were injected intraperitoneally (i.p.) with 4% thioglycolate (TLR2-deficient and TLR4-deficient mice were obtained from the Collaborative Innovation Center of Model Animal, Wuhan University). Peritoneal exudate cells were harvested 4 days later and identified by microscopic analysis and non-specific esterase staining. When >90% of the exudate cells were identified as macrophages, the cells were plated at a density of 10^6^ in 12-well plates.

### 2.3. Purification of the HP1717 Protein

The *hp1717* gene from the 05ZY genome was amplified using PCR. The primers are shown in Table 2. The cloned gene was inserted into the prokaryotic expression plasmid pET28a and transformed into the *Escherichia coli* BL21 strain. Isopropyl-b-d-thiogalactopyranoside (0.2 mM) was added to the cells to induce protein expression, and the culture was incubated for 4 h at 37 °C. Then, the recombinant protein was purified by Ni-NTA agarose chromatography. The endotoxin in the purified protein was removed using an Endotoxin Removal Kit (Genmed Scientifics Inc., WILMINGTON, DE, USA) and measured by a Quantitative Chromogenic Tachypleus Amebocyte Lysate for Endotoxin Detection Kit (Chinese Horseshoe Crab Reagent Manufactory Co., Ltd., Xiamen, China). Next, the protein was filtered with a 0.22 μm membrane and stored at −80 °C for subsequent experiments.

### 2.4. Flow Cytometry Analysis

Flow cytometry analysis was used to detect HP1717 on the surface of *S. suis* bacterial cells, as previously described [18]. Briefly, the overnight cultures were grown in TSB to an OD600 of 0.6. Bacteria were washed twice in PBS, and the cell density was adjusted to approximately 10^8^ CFU/mL. Bacterial suspensions were incubated with mouse anti-rHP177 or preimmune serum as control. After 1 h, cells were washed three more times with PBS and then incubated with goat anti-mouse IgG–fluorescein isothiocyanate (FITC) (KPL) for 1 h. This was followed by paraformaldehyde fixation for 30 min. Then, the samples were detected using a flow cytometer (Becton Dickinson, CA, USA).

### 2.5. RNA Extraction and qRT-PCR

RAW 264.7 cells were treated with 1 μg·mL^−1^ HP1717 or 100 ng·mL^−1^ lipopolysaccharide (LPS) for 6 h, and then the total RNA of the cells was extracted using the TRIzol^®^ reagent (Invitrogen, Paisley, UK). Complementary DNA was synthesized from 4 μg of RNA by using AMV reverse transcriptase (Takara, Dalian, China). qRT-PCR was performed using ViiA™ 7 Software (Applied Biosystems, Waltham, MA, USA) with the SYBR Green PCR Kit (Roche, Basel, Switzerland). All the primers used in qRT-PCR are listed in Table 2. The relative levels of target gene expression were normalized with the GAPDH housekeeping gene using the 2^−ΔΔCt^ method.

### 2.6. Examination of Cytokines by ELISA

The concentrations of IL-1β, MCP-1, and TNF-α in the cell culture supernatants or sera were determined using commercially available ELISA kits (BioLegend, San Diego, CA, USA) following the manufacturer’s instructions the detection limits of IL-1β, MCP-1, and TNF-α were 22, 20, and 8 pg⋅mL^−1^, respectively.

### 2.7. Determination of HP1717 Recognition Receptor

To investigate which receptor was specifically responsible for HP1717-mediated cytokine upregulation, we first detected the mRNA levels of TLR2 and TLR4 after HP1717 stimulation by qRT-PCR. Next, antibody blocking assays were performed to verify the results of qRT-PCR. Briefly, after pretreatment with 8 μg of an anti-TLR2 (BioLegend, San Diego, CA, USA) or anti-TLR4 (BioLegend) antibody for 30 min, RAW264.7 cells were incubated with 1 μg⋅mL^−1^ HP1717 for 6 h. The concentrations of IL-1β, MCP-1, and TNF-α in the culture supernatants were determined by ELISA. TLR2−/− and TLR4−/− macrophages isolated from TLR2−/− and TLR4−/− mice were also used to prove this result.

### 2.8. Analysis of the HP1717-Induced Cell Signal Transduction Pathway

For cell signaling analysis, 30 min prior to the addition of HP1717, RAW 264.7 cells were incubated with the following specific inhibitors: SB203580 (for p38 MAPK, 10 μM; Cayman Chemical, Michigan, USA), SP600125 (for JNK, 10 μM; Cayman Chemical), pyrrolidine dithiocarbamate (PDTC; for NF-κB, 20 μM; Sigma), LY294002 (for PI3K, 20 μM; Cayman Chemical), and U0126 (for ERK1/2, 10 μM; Cayman Chemical). After HP1717 stimulation for 6 h, the culture supernatants were collected for ELISA to measure IL-1β, MCP-1, and TNF-α. According to the conditions of cytokine expression in each group, we performed a preliminary screen for the signal transduction molecule induced by HP1717. To verify the above analysis results, we performed Western blotting to analyze the phosphorylation of signal transduction molecules activated by HP1717. Briefly, after being stimulated with 1 μg·mL^−1^ HP1717 for 6 h, RAW 264.7 cells were washed once with cold PBS and incubated on ice for 15 min using radioimmunoprecipitation assay lysis buffer with phosphatase inhibitors (Roche, Basel, Switzerland). The supernatants were collected, and their protein concentrations were quantified by the Bradford protein assay. Then, 20 μg of proteins were resolved on a 12% sodium dodecyl sulfate-polyacrylamide gel electrophoresis (SDS-PAGE) gel, followed by electrotransfer to a 0.22 μm nitrocellulose membrane. After blocking of the NC membrane by 1% BSA for 1 h at 25 ℃, these proteins were probed with specific antibodies against the phosphorylated forms of ERK1/2 and NF-κB p65 (Cell Signaling Technology, Beverly, MA, USA), and GAPDH was assessed as an internal control using anti-GAPDH antibody (PMK Biotechnology Co., Ltd, Wuhan, China). The bands were detected using a HRP-conjugated secondary antibody and an enhanced chemiluminescence (ECL) system (Amersham Life Science, Arlington Heights, IL, USA).

### 2.9. Mutant Construction

The deletion of HP1717 was performed as previously described [17]. The upstream and downstream homologous fragments of the *hp1717* gene were separately amplified using PCR. The two fragments were fused together using overlapping PCR and then digested using *Bam*H I and *Hin*dⅢ. The obtained fragment was inserted into the shuttle plasmid pSET4s and transformed into *E. coli* DH5α. After cloning, the recombinant plasmid was electroporated into 05ZY. The potential mutant strain was screened for spectinomycin resistance and thermosensitive suicide of the pSET4s vector. The cells were then examined using P1/P2 (to detect *gdh*), P3/P4 (to detect the pSET4s vector), P5/P6 (to detect *hp1717*), and P7/P8 (external primers to distinguish the size of the wild type and mutant).

### 2.10. Gram Staining and Transmission Electron Microscope (TEM) Observation

The Gram stain assay was carried out as previously described [19]. The cells were allowed to grow to an OD600 of 0.6 in THB at 37 °C, and Gram-stained cells were examined under a light microscope using oil immersion. TEM assays were performed in accordance with previously described methods [20]. Next, 05ZY and Δ1717 cells were harvested at OD600 of 0.6 and fixed with 2.5% glutaraldehyde overnight. The samples were then treated with 2% osmium tetroxide for 2 h and dehydrated by serial dilution using ethanol. The dehydrated cells were embedded in epoxy resin, and their morphological characteristics were observed using an H-7650 TEM (Hitachi, Tokyo, Japan).

### 2.11. Whole-Blood Bactericidal Assay

The whole-blood bactericidal experiment was performed as previously described [21]. First, 05ZY and mutant Δ1717 cells were cultured to mid-log phase (OD600 = 0.6). Next, 1 × 10^4^ CFU of 05ZY or an equal quantity of Δ1717 was added into 1 mL of whole blood for 2 h at 37 °C. At 0, 0.5, 1, and 2 h post-incubation, bacterial counts were determined. The initial bacterial volume was set to 100%, and the percentage of remaining bacteria was recorded at each time point. The assays were performed in triplicate and repeated three times.

### 2.12. In Vivo Experiments and Immunohistochemistry

This study was carried out in accordance with the recommendations of th·e Guide for the Care and Use of Laboratory Animals Monitoring Committee of Hubei Province, China, and the protocol was approved by the Committee on the Ethics of Animal Experiments at the College of Veterinary Medicine, Huazhong Agricultural University. For the in vivo experiments, 30 four-week-old male C57BL/6 mice were randomly divided into two groups, with 15 mice in each group. One group was intraperitoneally infected with 6 × 10^8^ CFU of 05ZY, and the second group received an equal quantity of Δ1717 by the same method. After 6 h, five mice in each group were sacrificed. The brains, lungs, livers, spleens, and kidneys were collected and fixed with 4% paraformaldehyde and sent to Hycell Biotechnology Company (Wuhan, China) for immunohistochemistry. Rehabilitation sera of pigs after infection with *S. suis* was used as a primary antibody (1:300), and HRP Goat Anti-Swine IgG (H+L) was used as the secondary antibody (1:200) (Abclonal, Wuhan, China). The disease onset and survival status in the remaining 20 mice were recorded for 1 week. In addition, 40 additional four-week-old female C57BL/6 mice were randomly assigned to two groups (20 mice per group) and challenged with a non-lethal dose (2 × 10^8^ CFU per mouse) of 05ZY, and the other group was given an equal quantity of the Δ1717 strain. At 3, 6, 9, and 12 h post-infection, five mice in each group were sacrificed to collect blood for ELISA detection of IL-1β, MCP-1, and TNF-α production.

### 2.13. Zymogram Analysis

Zymogram analysis was performed according to the previous research with some modifications [22]. 600 mL of *S. suis* grown to the mid-log phase was collected and washed twice with PBS and then dissolved in 10% SDS. After heating in boiling water for 30 min, the sample was placed overnight at room temperature to ensure adequate dissolution. After centrifuging at 16,500× *g* for 20 min, the supernatant was removed, and the precipitate was repeatedly washed with ddH_2_O to remove SDS and finally resuspended in 10 mL of ddH_2_O. Cells was added to a 12% SDS-PAGE resolving gel solution instead of water. BSA (negative control), Lysozyme (positive control) and HP1717 were loaded into each lane (samples were repeatedly loaded once for different staining), the running conditions of the gel were the same as those of the general PAGE gel (80V for concentrated gel and 120V for separation gel). The gel was washed with distilled water twice and incubated in ≈300 mL of refolding buffer (25 mM Tris-HCl and 1% (*v/v*) Triton X-100, pH 7.4) with gentle shaking at 37 °C for 12 to 14 h. The gel was divided into two parts and stained with Coomassie Brilliant Blue and Methylene Blue, respectively.

### 2.14. Statistical Analysis

Statistics for the survival assay were done with the log-rank test (Mantel–Cox). Other statistical analyses were performed by one-way or two-way ANOVA, followed by a multiple comparison. All experiments were performed at least three times, and data are expressed as the mean ± SD. A *p*-value <0.05 is considered significant. In the figures, * *p* < 0.05, ** *p* < 0.01, and *** *p* < 0.001. NS means no significance

## 3. Results

### 3.1. Characterization of *S. suis* 2 HP1717

Previous studies have indicated that the gene *SSU05_1717* in the genome of *S. suis* 2 Chinese strain 05ZYH33 encodes a periplasmic solute-binding protein (GenBank accession number: ABP90683) that consists of 365 amino acid residues [4]. Analysis of the 47 genomes of different isolates of *S. suis* that were completely sequenced in NCBI (https://www.ncbi.nlm.nih.gov/genome/genomes/199?) has shown that all of them contain *hp1717*, suggesting that this gene is widespread in *S. suis.* Further amino acid sequence homology analysis showed that HP1717 has certain similarities with many Gram-positive and negative bacteria. For example, the similarity between HP1717 and the homologous protein of *S.pneumoniae* (GenBank accession number: A0A0H2ZLQ1.1, Figure S1A) is 54%, and the similarity with the homologous protein of *S. pyogenes* (GenBank accession number: SQF37078.1; Figure S1B), *Staphylococcus aureus* (GenBank accession number: SUL86689.1; Figure S1C), *Bacillus vallismortis* (GenBank accession number: WP_121642973.1; Figure S1D), and *Escherichia coli* (GenBank accession number: NP_415615.1; Figure S1E) is 57%, 59%, 37%, and 27%, respectively.

### 3.2. HP1717 is Expressed on the Surface of *S. suis* 2

Previous studies have predicted HP1717 to be a secreted protein, but we did not find a signal peptide in our prior work [23]. To determine whether the protein was expressed on the cell surface of *S. suis 2*, we performed flow cytometry. As shown in Figure S2, the mean fluorescence intensity (MFI) of *S. suis 2* treated with mouse anti-HP1717 sera (B) was significantly higher than that of bacteria treated with preimmune serum (A). This indicates that HP1717 is not only expressed in 05ZY but also located on the bacterial cell surface.

### 3.3. HP1717 Induces Potent Expression of Pro-Inflammatory Cytokines in RAW264.7 Cells

**Figure 1:**
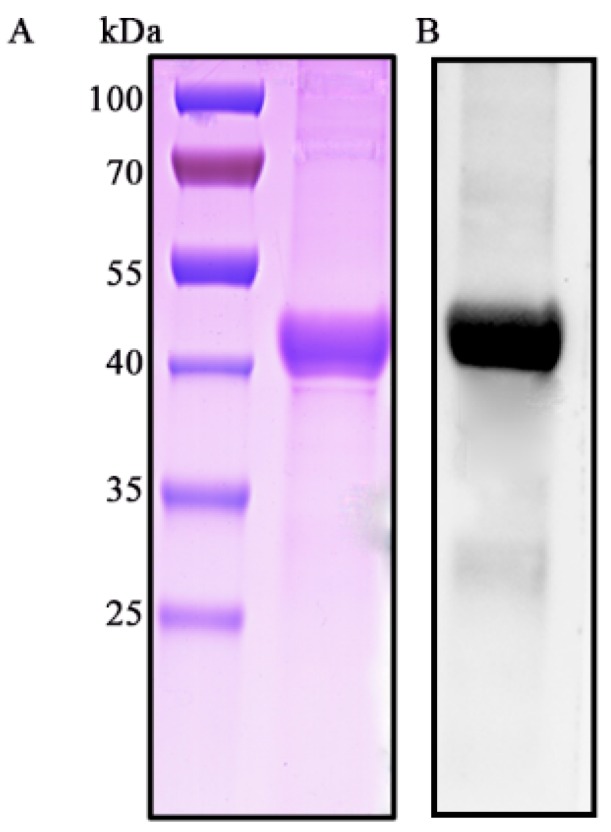
Purification of the recombinant HP1717 protein. (**A**) SDS-PAGE analysis. (**B**) Western blot analysis: the blot was probed with an anti-His tag monoclonal antibody (Cali-Bio).

**Figure 2:**
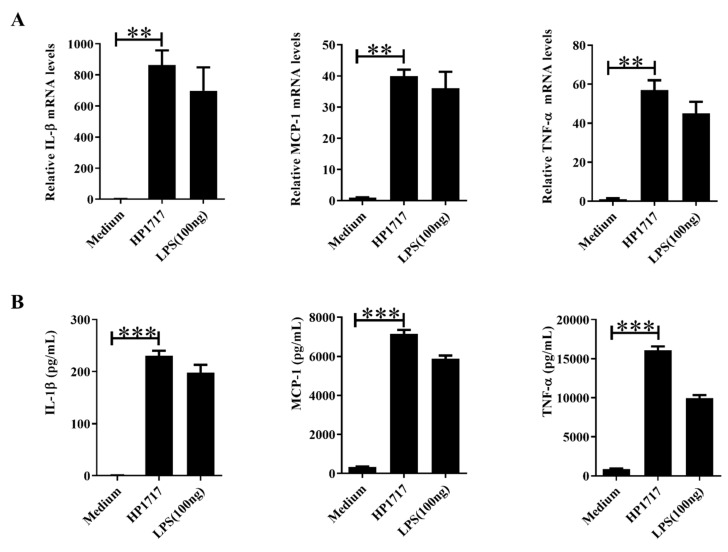
Induction of cytokine mRNA transcription and protein expression in RAW 264.7 macrophages after stimulation with recombinant HP1717. RAW 264.7 macrophages were treated with 100 ng·mL^−1^ lipopolysaccharide (LPS) (positive control), 1 μg·mL^−1^ HP1717 protein, or culture medium (negative control). (**A**) The cytokine mRNA levels were determined by qRT-PCR, and (**B**) the protein levels of IL-1β, MCP-1, and TNF-α in the culture supernatants were determined by enzyme-linked immunosorbent assay (ELISA). ** *p* < 0.01, *** *p* < 0.001.

HP1717 contains 365 amino acids and has an estimated molecular mass of approximately 40 kDa. SDS-PAGE (Figure 1A) and Western blotting (Figure 1B) analyses of the recombinant protein with an anti-His tag monoclonal antibody indicate that HP1717 was successfully expressed and purified. After removing endotoxins, the protein concentration was 2.5 mg·mL^−1^, and the endotoxin level was 0.02 EU/mL. Next, the results of both the quantitative real-time polymerase chain reaction (qRT-PCR) and the enzyme-linked immunosorbent assay (ELISA) analysis showed that HP1717 induced the massive expression of IL-1β, MCP-1, and TNF-α (Figure 2A,B), indicating that HP1717 led to a potent pro-inflammatory response in RAW264.7 cells.

### 3.4. The Pro-Inflammatory Activity of HP1717 is Dose-Dependent and Heat-Sensitive

**Figure 3:**
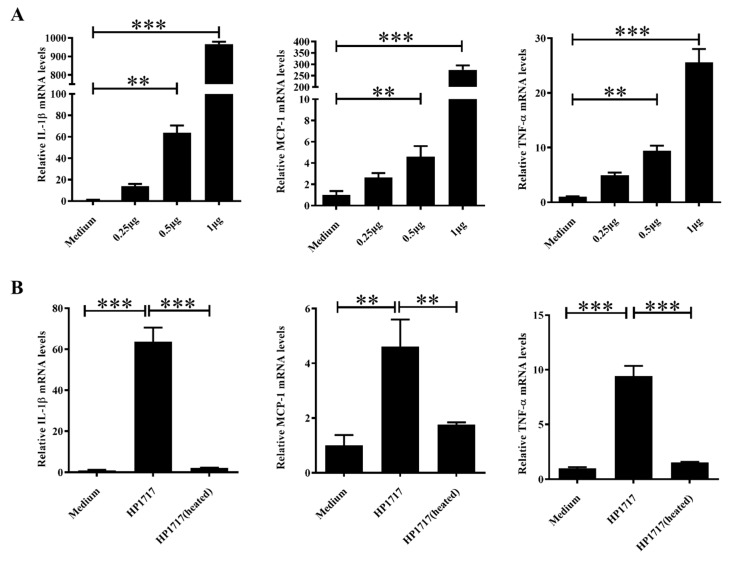
Experiments to determine the proinflammatory properties of HP1717. (**A**) The dose-dependent experiments were followed by qRT-PCR to measure transcript levels. (**B**) In the heat inactivation experiment, 1 μg·mL^−1^ HP1717 was inactivated by heating the protein at 100 °C for 10 min and then stimulating RAW264.7 cells with heat-inactivated HP1717. Then, the cytokine expression level was assessed by qRT-PCR. ** *p* < 0.01, *** *p* < 0.001.

To investigate whether the dose and activity of HP1717 affect its pro-inflammatory ability, the expressions of IL-1β, MCP-1, and TNF-α induced by different concentrations of HP1717 were measured by qRT-PCR (Figure 3A). The results show that higher concentrations of HP1717 induced higher levels of cytokine expression, indicating that the pro-inflammatory activity of HP1717 is dose-dependent. After pretreating the protein at 100 °C for 10 min [13], RAW264.7 cells were incubated with the heat-treated HP1717. We found that heat-treated HP1717 failed to stimulate the expression of IL-1β, MCP-1, and TNF-α, contrary to unheated HP1717 (Figure 3B). These findings indicate that the pro-inflammatory activity of HP1717 is heat-sensitive.

### 3.5. The Pro-Inflammatory Role of HP1717 Depends on the Recognition of TLR2

**Figure 4:**
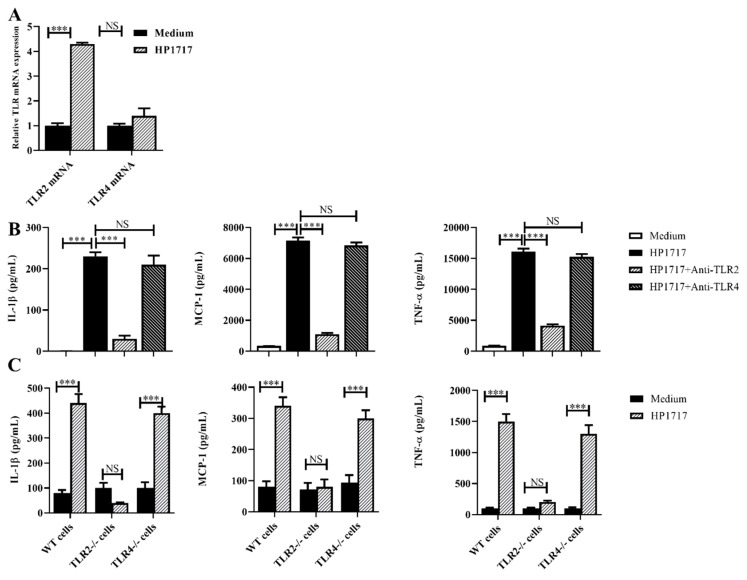
Recognition receptor of the HP1717-stimulated pro-inflammatory response. (**A**) After stimulation with HP1717, RAW264.7 cells were collected to analyze the mRNA levels of Toll-like receptor 2 (TLR2) or TLR4 by qRT-PCR. (**B**) Antibody blocking assays were conducted to determine the effects on cytokine expression. After pretreatment with 8 μg of anti-TLR2 or anti-TLR4 antibodies for 30 min, RAW 264.7 cells were incubated with 1 μg·mL^−1^ HP1717 for 6 h. The concentrations of IL-1β, MCP-1, and TNF-α were determined by ELISA. (**C**) After isolated from wild-type (WT), TLR2^−/−^ and TLR4^−/−^ mice. The primary peritoneal macrophages were incubated with 1 μg·ml^−1^ HP1717 for 6 h. The cytokine concentrations in the supernatants were determined by ELISA. *** *p* < 0.001; NS means no significant difference.

The qRT-PCR results show that TLR2 transcription was significantly upregulated in RAW264.7 cells stimulated by HP1717, but TLR4 transcription did not significantly change (Figure 4A). Antibody blocking experiments indicate that the pro-inflammatory effect of HP1717 almost disappeared when RAW264.7 cells were pretreated with the TLR2 antibody (Figure 4B). In addition, macrophages isolated from TLR2^−/−^ or TLR4^−/−^ mice were used to evaluate the pro-inflammatory activity of HP1717. The results indicated that HP1717 could stimulate significant pro-inflammatory responses in WT and TLR4^-/-^ macrophages, but not in TLR2^−/−^ macrophages (Figure 4C). Our results demonstrate that the pro-inflammatory ability of HP1717 depends on TLR2.

### 3.6. The Pro-Inflammatory Role of HP1717 Depends on the Phosphorylation of NF-κB and ERK1/2

**Figure 5:**
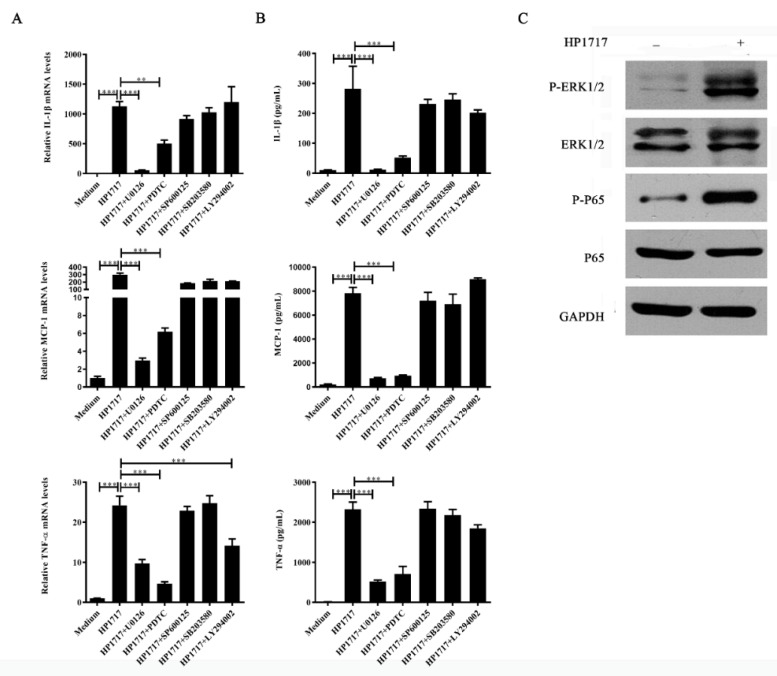
Signal transduction pathways of the HP1717-stimulated pro-inflammatory response in RAW 264.7 macrophages. After pretreatment with inhibitors of ERK1/2 (U0126), NF-κB (PDTC), JNK (SP600125), p38 MAPK (SB203580), or PI3K (LY294002) for 30 min, RAW 264.7 macrophages were stimulated with 1 μg·mL^−1^ HP1717 for 6 h. (**A**) The cytokine mRNA levels were determined by qRT-PCR, and (**B**) the protein levels of IL-1β, MCP-1, and TNF-α in the culture supernatants were determined by ELISA. (**C**) HP1717-induced phosphorylation of NF-κB and ERK 1/2 MAPK in RAW264.7 macrophages: RAW264.7 macrophages were stimulated with HP1717 (1 μg·mL^−1^) for 6 h. The cell lysates were analyzed by Western blotting using specific antibodies against P65, phospho-P65, ERK 1/2 MAPK, and phospho-ERK 1/2 MAPK. GAPDH was detected as an internal control using an anti-GAPDH antibody. The results shown are representative of three independent experiments. ** *p* < 0.01, *** *p* < 0.001.

To further investigate the pro-inflammatory mechanism of HP1717, we examined the HP1717-dependent signaling pathway. We used inhibitors of p38 mitogen-activated protein kinase (MAPK), c-Jun N-terminal kinase (JNK), NF-κB, phosphoinositide 3-kinase (PI3K), and ERK1/2 to identify the signaling pathways required for the pro-inflammatory response to HP1717. Our results show that U0126 and pyrrolidine dithiocarbamate (PDTC), which inhibit ERK1/2 and NF-κB, respectively, significantly suppressed the pro-inflammatory response to HP1717 (Figure 5A,B), suggesting that HP1717-induced cytokine expression probably depends on the phosphorylation of NF-κB and ERK1/2. Western blot analysis was performed to verify this hypothesis. We found that HP1717 markedly stimulated the phosphorylation of NF-κB and ERK 1/2 MAPK in RAW264.7 cells compared with the control group (Figure 5C). All of the above experiments demonstrate that the HP1717-induced cytokine expression is dependent on the phosphorylation of NF-κB and ERK1/2.

### 3.7. Construction and Characterization of Mutant Strain Δ1717

**Figure 6:**
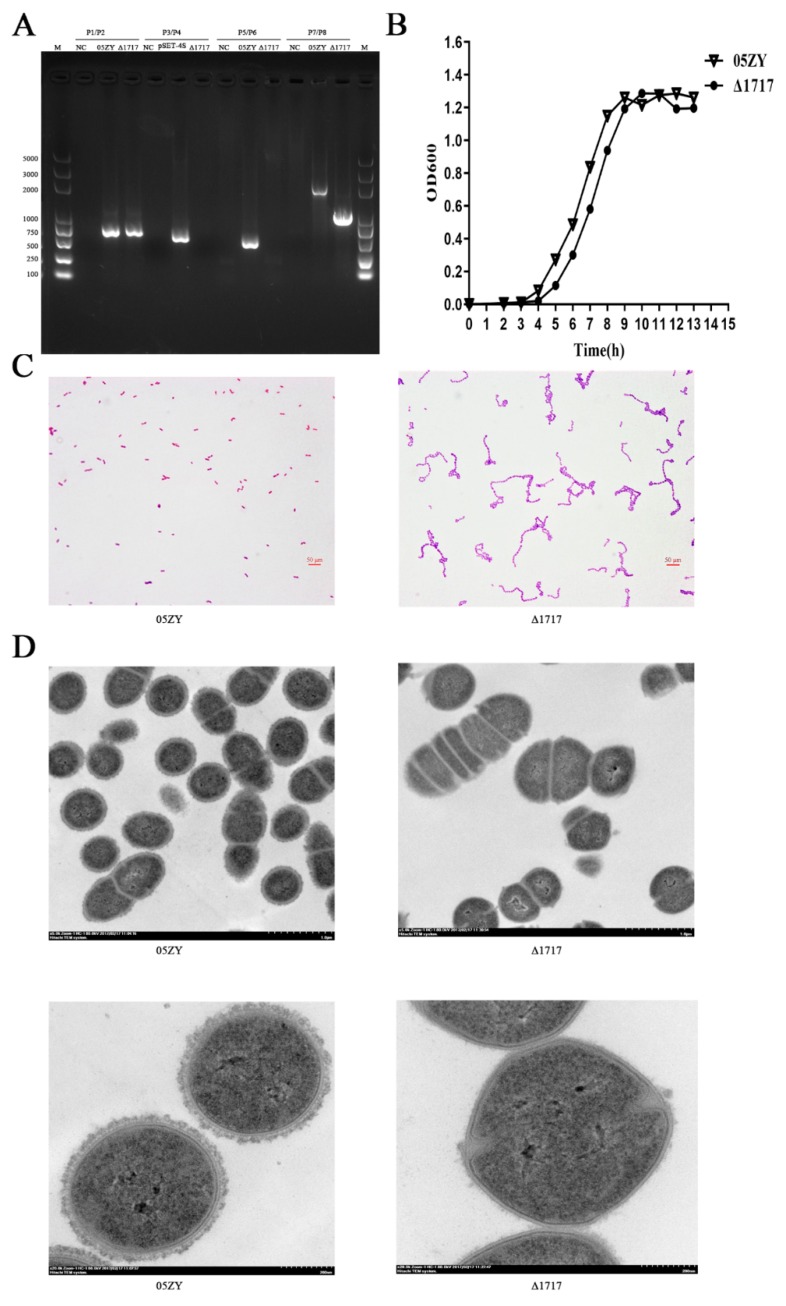
Construction and confirmation of the Δ1717 mutant. (**A**) Confirmation of the Δ1717 mutant by PCR using the primer pairs P1/P2 (to detect the *gdh* gene), P3/P4 (to detect the pSET4s vector), P5/P6 (to detect the *hp1717* gene), and P7/P8 (external primers of *hp1717* to distinguish the size of the wild type and mutant). (**B**) Growth curves of 05ZY and Δ1717: the bacteria were cultured in tryptic soy broth (TSB) containing 5% newborn bovine serum at 37 °C. The absorbance at 600 nm was measured at intervals of 1 h. The results shown are representative of three independent experiments. (**C**) Light microscope morphology of 05ZY and Δ1717 were observed by Gram staining (×1000). (**D**) The capsules of 05ZY and Δ1717 were detected by transmission electron microscopy (the top images are ×5000, and the bottom images are ×20,000).

The deletion of the *hp1717* gene was verified by polymerase chain reaction (PCR) analysis. As shown in Figure 6A, the *gdh* gene was detected in both 05ZY and Δ1717 genomes (using primers P1/P2). On the contrary, the internal fragment of the *hp1717* gene (439 bp) could be detected in 05ZY genome, but it was absent from the Δ1717 genome (using primers P5/P6). In addition, the pSET4s vector could not be amplified from the Δ1717 genome (using primers P3/P4). Furthermore, a large fragment (2071 bp) was obtained by amplifying the 05ZY genome with the external primers of *hp1717*, and a small fragment (973 bp) was obtained by amplifying the Δ1717 genome. In summary, we successfully deleted the *hp1717* gene. Next, we examined the phenotypes of Δ1717. The growth curve indicates that the growth of Δ1717 was slightly slower than that of 05ZY, but there were few differences between their curve peaks (Figure 6B). Interestingly, Gram staining results showed a significant increase in the chain length of Δ1717 (Figure 6C). To investigate whether CPS changed as a result of HP1717 deficiency, we examined the mutant and wild-type bacteria through transmission electron microscopy. Surprisingly, we found that the CPS of Δ1717 almost completely disappeared compared with that of 05ZY (Figure 6D). Since the significant change in the chain length of the mutant would lead to the inaccurate calculation of the mutant bacterial level by the conventional counting method (CFU counting), we used the absolute counting method on the basis of the 16S gene levels in the mutant and wild-type bacteria as quantification criteria by qRT-PCR. A standard curve was plotted using the absolute quantification of the known amount of T vector with the 16S gene, and we determined bacterial levels by measuring the 16S levels in Δ1717 and 05ZY. Bacterial levels in the subsequent experiments were determined by this method.

### 3.8. Δ1717 Displays Reduced Resistance to Whole-Blood Killing

**Figure 7:**
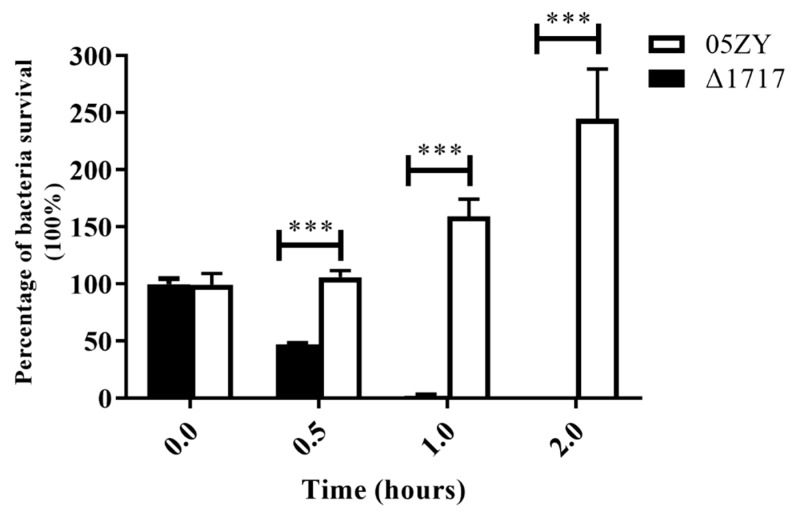
Whole-blood bactericidal experiment. First, 05ZY and Δ1717 were cultured to mid-log phase (OD600 = 0.6). Then 1 × 10^4^ CFU of 05ZY or an equal quantity of Δ1717 was added into 1 mL of whole blood for 2 h at 37 °C. At 0, 0.5, 1, and 2 h post incubation, bacterial counts were determined. The initial bacterial volume was set to 100%, and the percentage of remaining bacteria was recorded at each time point. The assays were performed in triplicate and repeated three times. *** *p* < 0.001.

The whole-blood sterilization assay was performed to determine the survival ability of Δ1717 in blood. The results show that the survival percentage of Δ1717 was significantly reduced compared with that of 05ZY at 0.5 and 1 h post-incubation, and the bacteria were completely cleared at 2 h (Figure 7), indicating that HP1717 may be involved in the resistance of the bacteria to whole-blood sterilization.

### 3.9. HP1717 Deficiency Leads to Attenuated Virulence, Decreased Pro-Inflammatory Ability, and Reduced Bacterial Loads in Mice

**Figure 8:**
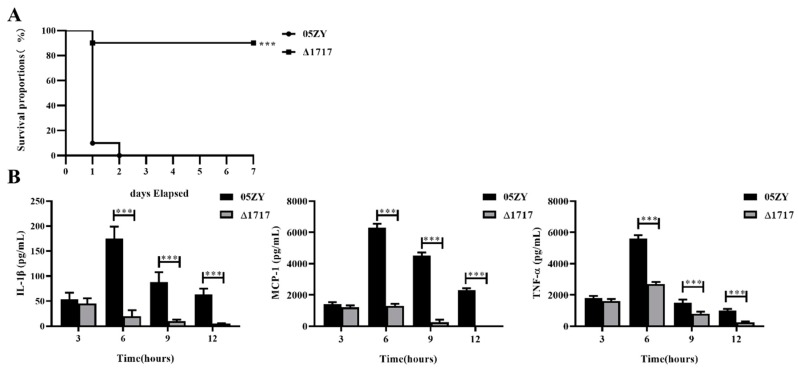
Mice infection and blood cytokine detection in an in vivo experiment. (**A**) Mice infection experiment. Female C57BL/6 mice in different groups were intraperitoneally infected with 6 × 10^8^ CFU of 05ZY or an equal quantity of Δ1717. The mortality of mice was recorded for 1 week. The results shown are representative of three independent experiments. (**B**) Blood cytokine detection in vivo experiment. Forty 4-week-old female C57BL/6 mice were randomly assigned to one of two groups (20 mice/group). One group was challenged with a non-lethal dose (2 × 10^8^ CFU per mouse) of 05ZY, and the other group was given an equal quantity of the Δ1717 strain. At 3, 6, 9, and 12 h post-infection, five mice in each group were sacrificed to collect blood for ELISA detection of IL-1β, MCP-1, and TNF-α production. *** *p* < 0.001.

**Figure 9:**
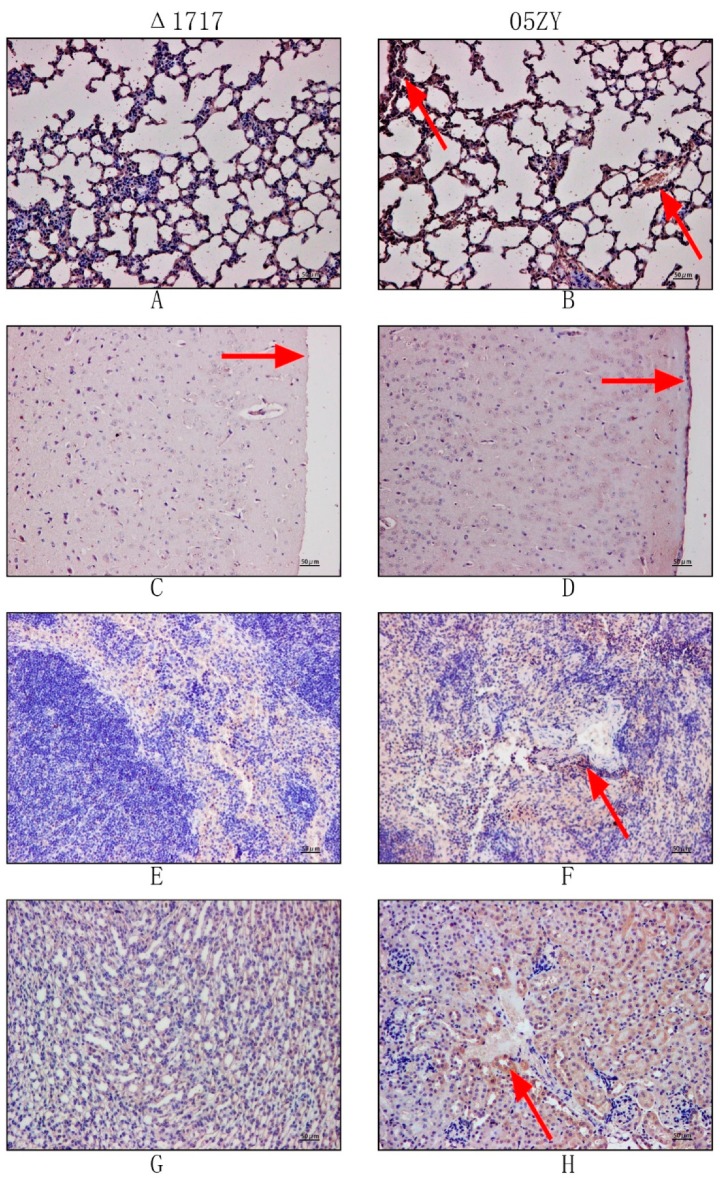
Immunohistochemistry of mouse organs after Δ1717 or 05ZY infection: 10 out of 30 (the remaining 20 were used for mice infection see Figure 8A) four-week-old male C57BL/6 mice were randomly divided into two groups, with 5 mice in each group. One group was intraperitoneally infected with 6 × 10^8^ CFU of 05ZY, and the other group was given an equal quantity of Δ1717. After 6 h, 5 mice in each group were sacrificed. The lungs, kidneys, spleens, and brains were extracted, fixed with paraformaldehyde for immunohistochemistry. The rehabilitation serum of pigs infected with 05ZY was used as the primary antibody for immunohistochemistry (1:300), and HRP Goat Anti-Swine IgG (H+L) was used as the secondary antibody (1:200) (Abclonal, Wuhan, China). (**A**,**B**) lungs; (**C**,**D**) meninges; (**E**,**F**) spleens; (**G**,**H**) kidney.

Mouse infection experiments were performed to investigate whether HP1717 influences the virulence of SS2. Because of the potential errors resulting from counting, we performed an absolute quantification experiment according to the above method. C57BL/6 mice were infected with 6 × 10^8^ CFU of 05ZY or an equal quantity of Δ1717. The mice infected with 05ZY died within 2 days. On the contrary, 90% of the mice infected with Δ1717 survived for 7 days post-infection and showed no obvious symptoms (Figure 8A). These results indicate that HP1717 deficiency significantly reduced the virulence of SS2 in these mice. The cause of the reduced virulence was investigated by determining the cytokine concentrations in the blood and the bacterial loads in different organs. The blood of the mice infected with Δ1717 contained lower concentrations of IL-1β, MCP-1, and TNF-α at 6, 9, and 12 h post-infection (Figure 8B). Results of the immunochemistry experiments show that the brown-yellow deposition in the lung, meninges, spleen, and kidney of the mice in the Δ1717 group was markedly lighter than that in the mouse organs in the 05ZY group (Figure 9), suggesting that the mice infected with Δ1717 contained lower bacterial loads in vivo. Finally, our results indicate that HP1717 may contribute to SS2 virulence by inducing high-level pro-inflammatory responses and influencing in vivo bacterial loads.

## 4. Discussion

Currently, SS2 remains a high threat to the global swine industry and human health [24]. Two large-scale outbreaks of STSLS in China provoked extensive public health concerns worldwide [25]. Although investigations of STSLS have been ongoing since SS2 was discovered, the current knowledge of the mechanism by which SS2 induces STSLS remains limited. Excessive inflammation and high bacterial loads are two important hallmarks of STSLS caused by SS2 infection [26,27], suggesting that clarification of the mechanisms of excessive inflammation and acute bacteremia resulting from SS2 infection would contribute to our understanding of SS2 pathogenesis, especially STSLS. In fact, the identification of some virulence-related factors has indeed enhanced our understanding of STSLS; for example, HP1330 can induce the pro-inflammatory response and influence in in vivo bacterial loads [13]. So, to further explain the mechanism of STSLS, it is necessary to identify additional molecules that can stimulate robust expression of pro-inflammatory cytokines and influence in in vivo bacterial loads. Our data demonstrate that HP1717 can induce a potent pro-inflammatory response in RAW264.7 cells. Furthermore, the deletion of HP1717 significantly reduces in vivo colonization of SS2. These findings suggest that HP1717 may be closely related to SS2-induced STSLS.

Generally, inflammation is beneficial to the host because it contributes to the clearance of the pathogen [28]. However, when an excessive inflammatory response is activated, the host can suffer serious damage [29]. A previous study indicated that, after SS2 infection, clearing the bacteria while not controlling excessive inflammation did not reduce the damage or death caused by SS2 infection [30], proving that an excessive inflammatory response is one of the important factors leading to pathological damage. In addition, as the most important virulence factor of *S. suis*, CPS can prevent host phagocytes from phagocytizing and killing bacteria [31,32], indicating that CPS is likely to be primarily responsible for immune evasion by *S. suis*. That is, excessive inflammation and the bacterial capsule are two of the most important factors of SS2 pathopoiesis. However, these two factors have not been considered at the same time in previous studies. Although CPS has been reported to induce the expression of several cytokines, high concentrations (at least 100 µg·mL^−1^ purified CPS) were required to observe its pro-inflammatory ability [33,34]. Furthermore, there has been little evidence that CPS is responsible for excessive inflammation. Our results demonstrate that HP1717 can induce robust expression of pro-inflammatory cytokines, and it achieves this at a very low dose (0.5 µg·mL^−1^; Figure 3A). At the same time, we also found that the deletion of HP1717 leads to almost the complete disappearance of CPS. Indeed, compared with the wild strain, the Δ1717 mutant displayed significantly reduced resistance to whole-blood killing and decreased in vivo bacterial loads. From the above results, we speculate that HP1717 can not only help SS2 escape or resist clearance by the host immune system by influencing the biosynthesis of CPS but also increase the SS2-induced pathological damage to the host by eliciting an excessive inflammatory response. Collectively, our data proved that HP1717 activity involves excessive inflammation and CPS at the same time. These findings lay the foundation for furthering our understanding of the pathogenesis of SS2.

Sequence homology analysis results suggest that HP1717 has certain similarity with MltG of *E. coli* and *Streptococcus pneumoniae* *(S.pneumoniae*) (Figure S1). Previous studies have shown that MltG has glycosyltransferase activity and can degrade peptidoglycan in *E. coli* [35]. But it didn’t have the effect of degrading peptidoglycan in *Streptococcus pneumoniae* [36]. In our study, it was found by zymogram experiments that HP1717 has no peptidoglycan degradation suggesting that the protein may be closer to the function of *S. pneumoniae* (Figure S3). However, in our study the phenotype of *S. suis* changed greatly after *hp1717* deletion. The chain length of the pathogen was significantly longer and the CPS almost disappeared, which was very different from *S. pneumoniae.* Therefore, further research of the specific function of HP1717 in *S. suis* needs to be performed in future.

We attempted to construct a complementary bacterial strain of Δ1717, but we were not successful (in most cases, it is difficult to do this with *S. suis*), as was also reported previously [18,37]. To evaluate whether the deletion of the *hp1717* gene influences the expression of its adjacent gene, we performed qRT-PCR. We did not detect any significant changes in the expression levels of *SSU05_1717*-adjacent genes (*SSU05_1716* and *SSU05_1718*) between 05ZY and Δ1717 (the results are not shown). To determine if any compensatory mutations might have occurred that could contribute to the phenotypes, whole genome sequence of the two strains were performed. The results showed that no meaningful compensatory mutations occurred in other locations, and genome sequencing and assembly data were uploaded to the Sequence Read Archive (SRA) under BioProject name PRJNA578680. In addition to CPS, we found that the chain length of SS2 is also significantly influenced by the deletion of HP1717. The chain length of Δ1717 increases by about 25 times compared with that of 05ZY, implying that the accuracy of bacterial counting would be highly erroneous using the conventional plate-counting method to compare the CFU count between 05ZY and Δ1717. To solve this problem, we inserted the 16S housekeeping gene of *S. suis* into a vector. After concentrations were measured, the recombinant vectors were used to draw a standard curve. The 16S levels in 05ZY and Δ1717 were measured by absolute quantitative PCR, and then the corresponding bacterial levels were calculated. Using this method, we performed all bacterial quantitation of 05ZY and Δ1717 before in vivo and in vitro experiments.

In conclusion, we identified HP1717 as a novel virulence-related factor of SS2 that can induce a potent pro-inflammatory response and influence the biosynthesis of CPS. Furthermore, our data demonstrated that the pro-inflammatory activity of HP1717 depends on the recognition of TLR2 and the phosphorylation of NF-κB and ERK1/2. These findings further expand our understanding of the pathogenesis and STSLS due to SS2.

## Figures and Tables

**Table 1:** Summary of the bacterial strains and plasmids used in this study.

Group	Name	Characteristics and Functions	Sources or References
Strains			
	05ZY	*Streptococcus suis* serotype 2 wild type	Laboratory collection
	Δ1717	Δ1717-deletion mutant strain	This study
	*Escherichia coli* DH5α	Cloning host for recombinant vector	TIANGEN
	*E. coli* BL21	Expression host for recombinant protein	TIANGEN
Plasmids			
	pSET4s	*E. coli*–*S. suis* shuttle vector; Spcr	Laboratory collection [17]
	pET28a	Expression vector; Kan	TIANGEN [13]

**Table 2:** Oligonucleotide primers used in this study.

Primers	Primers Sequence (5′-3′)	Functions or PCR Product
hp1717-1	CCCGAATTCATGTCGATTGTTGTAGTGGCA(EcoRI)	For amplification of the *hp1717* ORF gene
hp1717-2	CCCAAGCTTTTACTCATTATTAAGATGTGCATTTAC(HindIII)	
hp1717L1	CGCGGATCCCTACTGGGTTGTCGGTGGT(BamHI)	Upstream border of *hp1717*
hp1717L2	AACACATTGTCTCTGTTATCATTCTTAAAACAAAATTATGTGGTT	
hp1717R1	GATAACAGAGACAATGTGTTTAGCGGCATTATCTTGTTTT	Downstream border of *hp1717*
hp1717R2	CCCAAGCTTTAAGGGACAGGGAGTGGG(HindIII)	
MCP-1-1	AGAAGGAATGGGTCCAGACATA	For qRT-PCR assay
MCP-1-2	GTGCTTGAGGTGGTTGTGGA	
TNF-α-1	GAGTGACAAGCCTGTAGCCC	For qRT-PCR assay
TNF-α-2	GACAAGGTACAACCCATCGG	
IL-1β-1	TCATTGTGGCTGTGGAGAAGC	For qRT-PCR assay
IL-1β-2	TCATCTCGGAGCCTGTAGTGC	
GAPDH-1	TGGCCTTCCGTGTTCCTAC	For qRT-PCR assay
GAPDH-2	TGAAGTCGCAGGAGACAACC	
P1	TGGAAATGTTCAAGTCAACC	For PCR to detect the gdh
P2	CGTTTTTCTTTGATGTCCAC	
P3	CTACGAACTGCTAACA	For PCR to detect the pSET4s
P4	GAATACATACGAACAAAT	
P5	CCAAAGATGCCAAGGT	For PCR to detect the *hp1717*
P6	ATCGCCAAAGCACTTC	
P7	ATTCGTGGATTACCTG	External primers of *hp1717* ORF
P8	TACCATCAAGCTCGTC	
16s-1	CAGAAAGGGACGGCTAA	For amplification of the 16s gene
16s-2	CGGCTGGCTCCTAAAA	
16s-rt-1	AGATGGACCTGCGTTGTATT	For qRT-PCR assay
16s-rt-2	TCCGAAAACCTTCTTCACTC	
1717rt-1	TGGCATCTTTGGTTGA	For qRT-PCR assay
1717rt-2	CGAAAGGCTTGGACTA	

Underlined nucleotides denote enzyme restriction sites; Double-underlined nucleotides denote overlapping sequences.

## Supplementary Materials

Supplementary materials can be found at https://www.mdpi.com/2076-2607/7/11/522/s1. Figure S1: Homology comparison between HP1717 and different bacterial homologous proteins; Figure S2: Cell surface localization confirmed by flow cytometry; Figure S3: Zymogram analysis. SDS-PAGE were stained by Coomassie Brilliant Blue (left) and Methylene Blue (right) separately.
