# Supplementary material for: HP1717 Contributes to Streptococcus suis Virulence by Inducing an Excessive Inflammatory Response and Influencing the Biosynthesis of the Capsule
Source: Microorganisms. 2019 Nov 3;7(11):522. doi: 10.3390/microorganisms7110522 (PMC6920816; doi:10.3390/microorganisms7110522)
Supplement: Supplementary file 1 [file microorganisms-07-00522-s001.pdf]

A

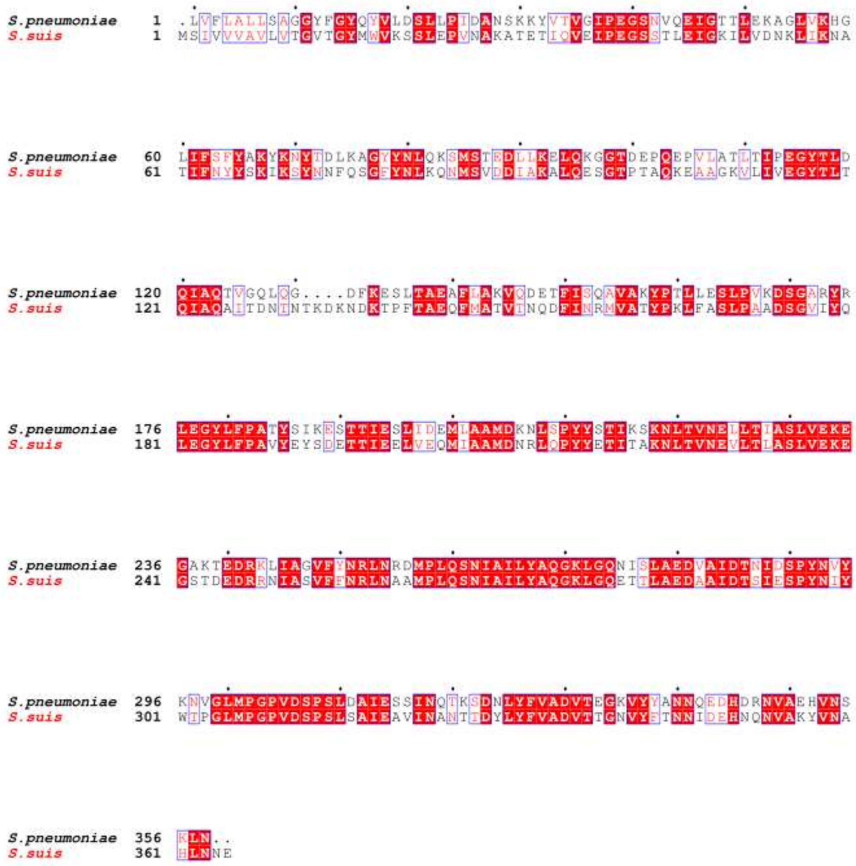
S.pneumoniae 1 .LVFLALLSAGGYFGYQYVLDSLLPIDANSKKYVTVGIPEGSNVQEIGTTLEKAGLVKHG
S.suis 1 MSIVVVAVLVTGVTGYMWVKSSLEPVNAKATETIQVEIPEGSSTLEIGKILVDNKLIKNA
S.pneumoniae 60 LIFSFYAKYKNYTDLKAGYYNLQKSMSTEDLLKELQKGGTDEPQEPVLATLTIPEGYTLD
S.suis 61 TIFNYYSKIKSYNNFQSGFYNLKQNMSVDDIAKALQESGTPTAQKEAAGKVLIVEGYTLT
S.pneumoniae 120 QIAQTVGQLQG....DFKESLTAEAFLAKVQDETFISQAVAKYPTLLESLPVKDSGARYR
S.suis 121 QIAQAITDNINTKDKNDKTPFTAEQFMATVTNQDFINRMVATYPKLFASLPAADSGVIYQ
S.pneumoniae 176 LEGYLFPATYSIKESTTIESLIDEMLAAMDKNLSPYYSTIKSKNLTVNELLTIASLVEKE
S.suis 181 LEGYLFPAVYEYSDETTIEELVEQMIAAMDNRLQPYYETITAKNLTVNEVLTLASLVEKE
S.pneumoniae 236 GAKTEDRKLIAGVFYNRLNRDMPLQSNIAILYAQGKLGQNISLAEDVAIDTNIDSPYNVY
S.suis 241 GSTDEDRRNIASVFFNRLNAAMPLQSNIAILYAQGKLGQETTLAEDAAIDTSIESPYNIY
S.pneumoniae 296 KNVGLMPGPVDSPSLDAIESSINQTKSDNLYFVADVTEGKVYYANNQEDHDRNVAEHVNS
S.suis 301 WTPGLMPGPVDSPSLSAIEAVINANTTDYLYFVADVTTGNVYFTNNIDEHNQNVAKYVNA
S.pneumoniae 356 KLN..
S.suis 361 HLNNE

B

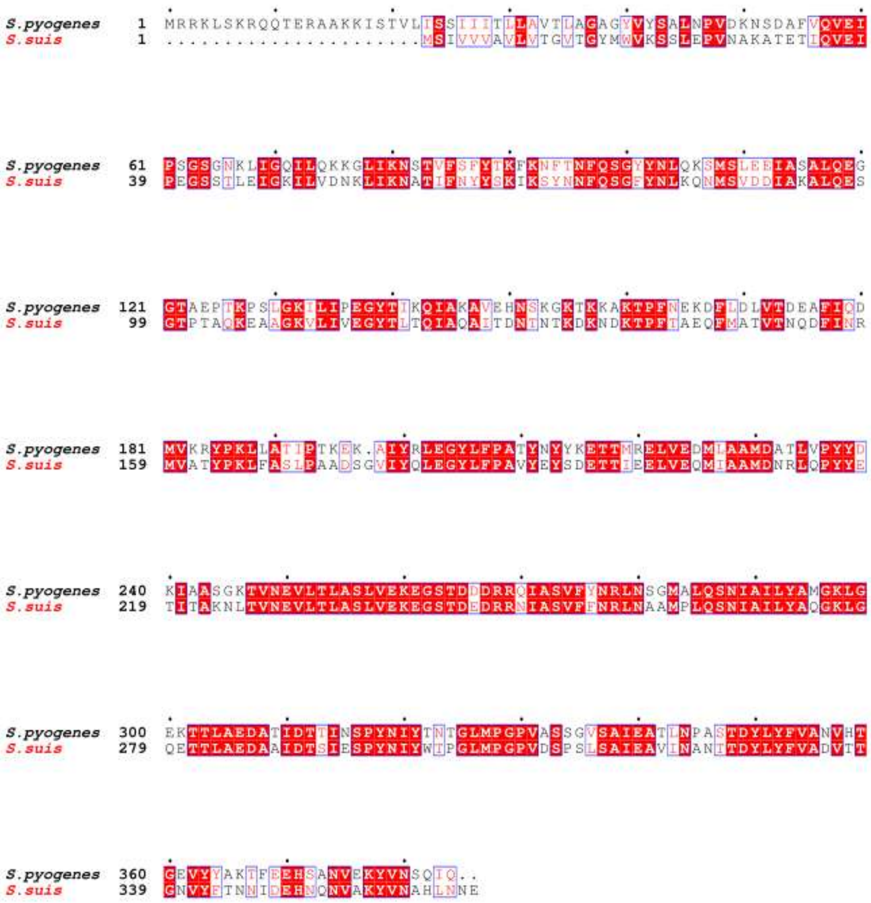
S.pyogenes 1
S.suis 1
S.pyogenes 61
S.suis 39
S.pyogenes 121
S.suis 99
S.pyogenes 181
S.suis 159
S.pyogenes 240
S.suis 219
S.pyogenes 300
S.suis 279
S.pyogenes 360
S.suis 339

C

```
Staphylococcus aureus   1   ...........GLAGTVYVYSALNPVDKNSKEFVQVEIPSGSGNKLIGQILQKEGLIKNS
S.suis                  1   MSIVVVAVLVTGVTGYMWVKSSLEPVNAKATETIQVEIPEGSSTLEIGKILVDNKLIKNA

Staphylococcus aureus  50   TVFSFYTKFKNFTNFQSGYYNLQKNMSLEEIAKALQEGGTAEPTKPALGKILIPEGYTIK
S.suis                 61   TIFNYYSKIKSYNNFQSGFYNLKQNMSVDDIAKALQESGTPTAQKEAAGKVLIVEGYTLT

Staphylococcus aureus 110   QIAKAVEHNSKGKDQKAKTPFHEKDFLNLVADETFIQKMVKKYPRLLGSIPTKEA.AVYR
S.suis                121   QIAQAITDNTNTKDKNDKTPFTAEQFMATVTNQDFINRMVATYPKLFASLPAADSGVIYQ

Staphylococcus aureus 169   LEGYLFPATYNYYEETTLESLIDDMLAATDATLAPYYDQIAASGKSVNDVLTLASLVEKE
S.suis                181   LEGYLFPAVYEYSDETTIEELVEQMIAAMDNRLQPYYETITAKNLTVNEVLTLASLVEKE

Staphylococcus aureus 229   GSTDDDRRQIASVFYNRLNNGMALQSNIAILYAMGKLGQKTTLAEDAAIDTTINSPYNIY
S.suis                241   GSTDEDRRNIASVFFNRLNAAMPLQSNIAILYAQGKLGQETTLAEDAAIDTSIESPYNIY

Staphylococcus aureus 289   TNTGLMPGPVDSSGLSAIEATINPASTDYLYFVANVHTGEVYYAKTFEEHSANVEKYVNS
S.suis                301   WTPGLMPGPVDSPSLSAIEAVINANTTDYLYFVADVTTGNVYFTNNIDEHNQNVAKYVNA

Staphylococcus aureus 349   QI...
S.suis                361   HLNNE
```

D

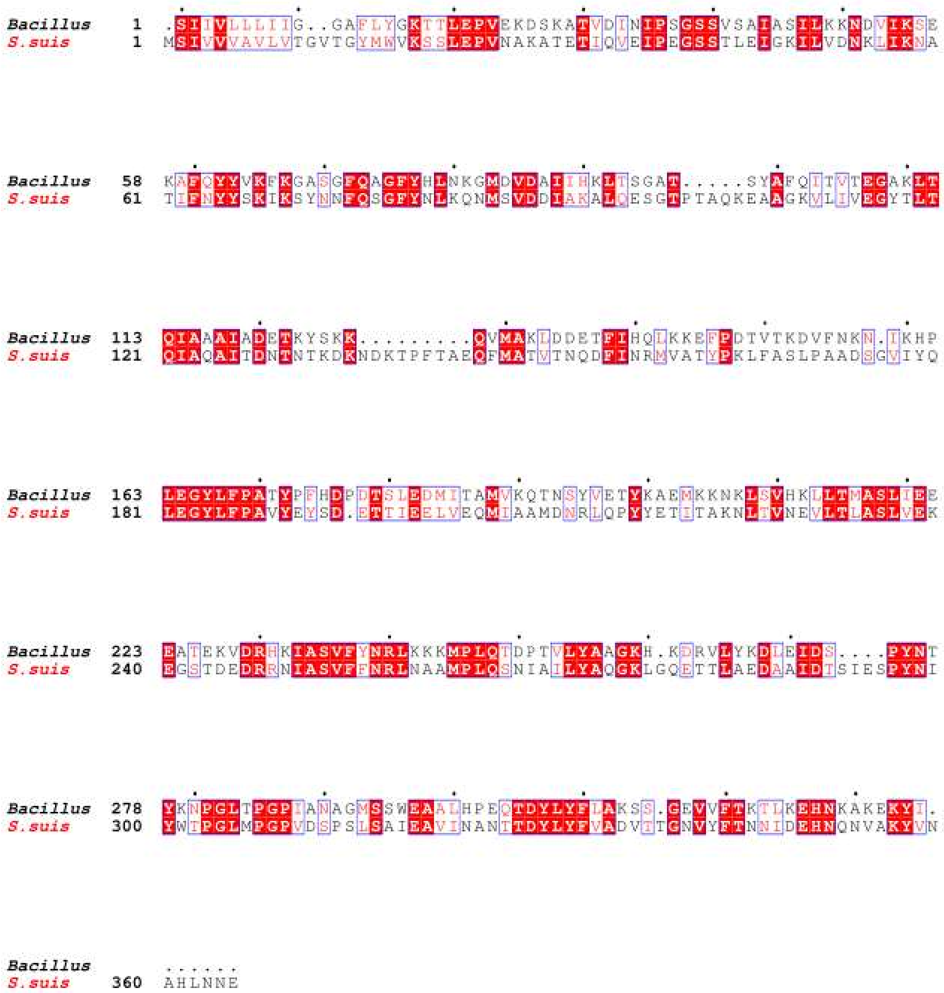
Bacillus 1 .SIIVLLLIIG..GAFLYGKTTLEPVEKDSKATVDINIPSGSSVSAIASILKKNDVIKSE
S.suis 1 MSIVVVAVLVTGVTGYMWVKSSLEPVNAKATETIQVEIPEGSSTLEIGKILVDNKLIKNA
Bacillus 58 KAFQYYVKFKGASGFQAGFYHLNKGMDVDAIIHKLTSGAT.....SYAFQITVTEGAKLT
S.suis 61 TIFNYYSKIKSYNNFQSGFYNLKQNMSVDDIAKALQESGTPTAQKEAAGKVLIVEGYTLT
Bacillus 113 QIAAAIADETKYSKK.........QVMAKLDDETFIHQLKKEFPDTVTKDVFNKN.IKHP
S.suis 121 QIAQAITDNTNTKDKNDKTPFTAEQFMATVTNQDFINRMVATYPKLFASLPAADSGVIYQ
Bacillus 163 LEGYLFPATYPFHDPDTSLEDMITAMVKQTNSYVETYKAEMKKNKLSVHKLLTMASLIEE
S.suis 181 LEGYLFPAVYEYSD.ETTIEELVEQMIAAMDNRLQPYYETITAKNLTVNEVLTLASLVEK
Bacillus 223 EATEKVDRHKIASVFYNRLKKKMPLQTDPTVLYAAGKH.KDRVLYKDLEIDS....PYNT
S.suis 240 EGSTDEDRRNIASVFFNRLNAAMPLQSNIAILYAQGKLGQETTLAEDAAIDTSIESPYNI
Bacillus 278 YKNPGLTPGPIANAGMSSWEAALHPEQTDYLYFLAKSS.GEVVFTKTLKEHNKAKEKYI.
S.suis 300 YWTPGLMPGPVDSPSLSAIEAVINANTTDYLYFVADVTTGNVYFTNNIDEHNQNVAKYVN
Bacillus ......
S.suis 360 AHLNNE

E

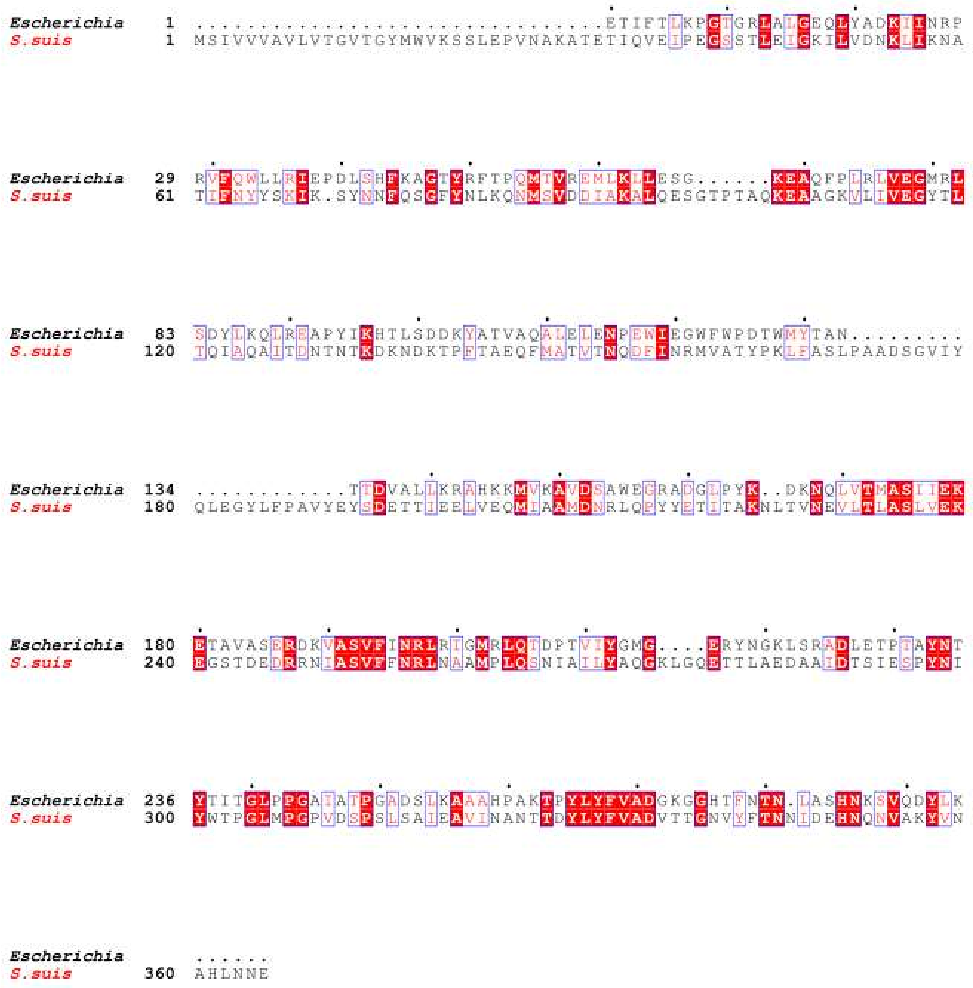

Figure S1. Homology comparison between HP1717 and different bacterial homologous proteins. HP1717 is shown in red font.(A) *Streptococcus pneumoniae* (A0A0H2ZLQ1.1)(B) *Streptococcus pyogenes* (SQF37078.1) (C) *Staphylococcus aureus* (SUL86689.1) (D) *Bacillus vallismortis* (WP_121642973.1) (E) *Escherichia coli* (NP_415615.1). The image was generated using the program ESPript 3.0 after the raw data were processed using ClustalW2.

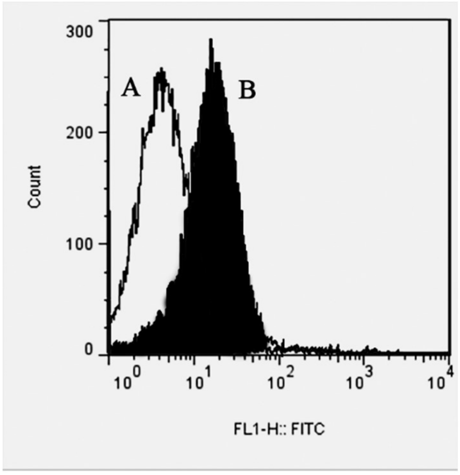

Figure S2. Cell surface localization confirmed by flow cytometry. Briefly, overnight cultures were pelleted, washed with PBS, and adjusted to 10^8 CFU/mL. The bacteria were then incubated with mouse anti-rHP177(B) or preimmune serum (A) as a control for 1 h. Following three washes, the cells were incubated with goat anti-

mouse IgG–fluorescein isothiocyanate (FITC) (KPL) for 1 h and then fixed with 4% paraformaldehyde for 30 min. After washing with PBS, the cells were analyzed by flow cytometry.

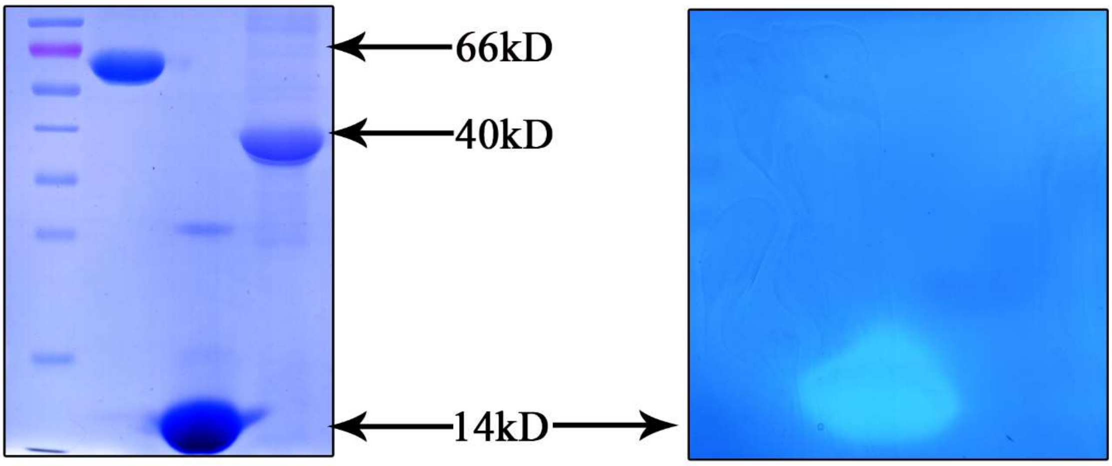

Figure S3. Zymogram analysis. SDS-PAGE were stained by Coomassie Brilliant Blue (left) and Methylene Blue (right) separately. Methylene blue can dye peptidoglycan and the light bands is clearing zones showing degradation of the PG of *S. suis* incorporated in the SDS–PAGE gel. M: Mark; L1: BSA; L2 Lysozyme:; L3: HP1717.
